# Diagnostic characteristics of the Rapid ESBL NP^®^ test and ResistanceFinder 2SMART multiplex PCR for the detection of ESBL directly on urine samples

**DOI:** 10.1007/s10096-025-05256-9

**Published:** 2025-09-25

**Authors:** Marit W. Bot, Jacky Flipse, Sabien L.M. Bongers, Chiara Giardino, Lieke E.K.W. Geraets, Antoinette A.T.P. Brink, Jacobien J. Hoogerwerf, Jaap ten Oever, Heiman F.L. Wertheim, Maurits P.A. van Meer

**Affiliations:** 1 Department of Medical Microbiology and Immunology, Rijnstate Hospital, Arnhem, the Netherlands; 2 Department of Medical Microbiology, Radboud University Medical Center, Nijmegen, the Netherlands; 3 Laboratory for Medical Microbiology and Immunology, Dicoon, Elst, the Netherlands; 4 PathoFinder B.V., Randwycksingel 45, Maastricht, 6229 EG Netherlands; 5 Department of Internal Medicine, Radboud University Medical Center, Nijmegen, the Netherlands; 6 Radboud Community for Infectious Diseases, Radboud University Medical Center, Nijmegen, the Netherlands

**Keywords:** Rapid diagnostics, Extended-spectrum beta lactamase, Urinary tract infection, Phenotypic detection, PCR

## Abstract

**Purpose:**

To determine the diagnostic characteristics of the phenotypic Rapid ESBL NP^®^ test and a prototype version of the molecular ResistanceFinder 2SMART multiplex PCR for the rapid detection of ESBL in patients with urinary tract infections.

**Methods:**

The Rapid ESBL NP^®^ test and the ResistanceFinder 2SMART multiplex PCR were performed on direct urine samples (enterobacterales, ≥10^5^ CFU/ml and ≥ 3 plusses of leukocytes in Gram stain) collected at Rijnstate and Radboudumc and compared to conventional urine cultures. The sensitivity, specificity, positive predictive value (PPV) and negative predictive value (NPV) were calculated with 95% confidence intervals.

**Results:**

In total, 218 urine samples were collected, of which 66 contained ESBL-producing bacteria, 102 contained Gram-negative bacteria without ESBL production and 50 contained no clinically significant growth. The Rapid ESBL NP^®^ test gave 16 (7.3%) non-interpretable results. After excluding the non-interpretable results, the test had a sensitivity, specificity, PPV and NPV of 96.8% (89.0-99.6), 100% (97.4–100), 100% (94.1–100) and 98.6% (94.7–99.6), respectively. Using detection of CTX-M and VEB genes as positive, the ResistanceFinder 2SMART multiplex PCR had a sensitivity, specificity, PPV and NPV of 95.5% (87.3–99.1), 94.1% (89.1–97.3), 87.5% (78.8–93.0) and 98.0% (94.0-99.3), respectively.

**Conclusion:**

The NPVs for both tests were high. However, the lower PPV of the ResistanceFinder2 SMART multiplex PCR could lead to increased overuse of carbapenems when implemented into clinical practice. The Rapid ESBL NP^®^ test could be a suitable screening tool for ESBL-producing bacteria in clinical urine samples, ensuring more accurate prescription of antibiotics, though prospective validation is necessary first.

## Introduction

Resistance to antibiotics, particularly in Gram-negative bacteria, is a major threat to patients as the subsequent failure to treat infections is a leading cause of death [1]. A large patient group affected by antibiotic resistant infections are those with (complicated) urinary tract infections (cUTI), defined in this study as having 1–2 species of enterobacterales, a CFU of at least 10^5^/mL and 3–4 plusses of leukocytes in the Gram stain. In standard clinical practice, patients with an indication for hospital admission are treated empirically with a second- or third-generation cephalosporin based on regional antibiotic resistance prevalence. However, if a patient is suspected to carry extended spectrum beta-lactamase (ESBL) or other multidrug-resistant Gram-negative bacteria, the choice of antibiotic treatment is often a carbapenem, or the addition of a potentially nephrotoxic aminoglycoside. As the prevalence of ESBL producers has increased in recent years in the Netherlands, so has the prescription of carbapenems [2]. Unfortunately, incorrect- and overuse of carbapenems contribute to their antibiotic resistance. Traditional culture-based techniques to determine the antibiotic susceptibility pattern take several days. Rapid antibiotic resistance tests (RATs) are promising as an early diagnostic method to prevent misuse of antibiotics in the first days of antibiotic treatment [3–5].

RATs can be roughly divided into two categories: phenotypical and genotypical. An example of a phenotypical RAT is the Rapid ESBL NP^®^ test (Liofilchem, Roseto degli Abruzzi, Italy). This test reports a sensitivity and specificity of 93.9% and 98.5%, respectively, for ESBL detection when used on bacterial isolates [6]. A recent study demonstrated the potential of using the Rapid ESBL NP^®^ test directly on urine samples [7]. The ResistanceFinder 2SMART multiplex PCR (PathoFinder, Maastricht, the Netherlands), currently at the prototype stage, is an example of a genotypical RAT. The PCR is able to detect various resistance genes in clinical samples. In this study, we compared the performance of the Rapid ESBL NP^®^ test and the ResistanceFinder 2SMART multiplex PCR directly on urine samples. RAT results were compared to the routine urine cultures and subsequent antimicrobial susceptibility testing (AST) results with possible confirmatory phenotypical testing, if suspected for ESBL. We chose to compare the results of the RATs to this gold standard as this represents the routine clinical practice on which antibiotic treatment is based.

## Materials and methods

This retrospective study used refrigerated urine samples from patients presenting to two Dutch hospitals, Rijnstate and Radboudumc, between September 2023 and January 2024 to assess the performance of two RATs, the Rapid ESBL NP^®^ test and the ResistanceFinder 2SMART multiplex PCR, compared to the gold standard of culture-based AST results with possible phenotypical ESBL confirmatory testing. During five months, the medical microbiology laboratory information systems were searched for positive urine cultures with one or two Enterobacterales species having growth of ≥ 10^5^ CFU/ml and 3–4 plusses of leukocytes in Gram stain. These species were both third generation cephalosporin susceptible (i.e., ESBL negative) and phenotypically confirmed as positive for ESBL production. The corresponding urines were retrieved and 50 urine samples which did not have clinically relevant bacterial growth were added. We defined no clinically relevant growth as no bacterial growth or urogenital flora without suspicion for uropathogens. The inclusion of ESBL negative, but culture positive samples, was halted after including 102 samples to balance the data.

All urine samples were cultured and when positive, AST was performed on each isolate with semi-automatic testing by Vitek2 (bioMérieux, Marcy-l’Étoile, France) in Rijnstate and Phoenix (BD, Franklin Lakes, USA) in Radboudumc. An elevated MIC of > 1 mg/L of cefotaxime and/or ceftazidime [8] prompted subsequent ESBL confirmatory testing. Confirmation of ESBL production was carried out by the ESBL combination disc test using disc diffusion of the combinations of cefotaxime, ceftazidime and cefepime alone versus these three cephalosporins with clavulanic acid (ESBL screen disk kit, Liofilchem, Roseto degli Abruzzi, Italy). Confirmation is positive for ESBL when a ≥ 5 mm increase of inhibition zone diameter is measured for the discs containing a cephalosporin together with clavulanic acid.

### Rapid ESBL NP^®^ test

The Rapid ESBL NP^®^ test detects the presence of ESBL through the hydrolysis of the beta-lactam ring of cefotaxime, which causes a decrease in pH, visualised by a phenol red pH indicator [9]. For direct urine samples, we adapted the protocol for the Rapid ESBL NP^®^ test based on Dortet et al. [10]. First, 4.5mL of urine was centrifuged for 10 min at 2560 rpm. Supernatant was discarded and the pellet was dissolved in 1.5mL of distilled water. The resulting suspension was centrifuged again, and the supernatant was discarded. From here, we were able to follow the instructions for use provided by the manufacturer, using the washed pellet instead of a 10 µl full loop of bacterial colonies, and using 300 µl of the supplied lysis buffer instead of 400 µl. During the study period, the Rapid ESBL NP^®^ tests were performed once a week. Samples were stored in the fridge until the test was performed. The average time between samples collection and performing the Rapid ESBL NP^®^ test was 6.1 days (range: 2–16 days).

### ResistanceFinder 2SMART multiplex PCR

The ResistanceFinder 2SMART multiplex PCR identifies various resistance genes using the 2SmartFinder technology by PathoFinder [11], such as ESBL, (plasmid) AmpC, carbapenemase and colistin resistance genes. In this study, we focussed on the detection of ESBL genes, namely CTX-M, SHV, TEM, VEB, PER and BEL. The 2SmartFinder protocol consists of a multiplex amplification step and a 2SMART step. In the 2SMART step, specialised primers and universal primers direct further amplification, internal fluorescent labelling and incorporation of detection sequences (“stuffers”). The stuffer sequences have varying melting temperatures. Hybridisation of probed to their respective stuffer sequence, followed by melting curve analysis, will reveal which pathogen was present in the sample. The prototype version of the ResistanceFinder 2SMART multiplex PCR does not differentiate between the various SHV and TEM subtypes encoding for ESBL activity and subtypes not associated with ESBL activity.

Briefly, 200 µl of urine was extracted using NucliSens easyMag (bioMérieux, Marcy-l’Étoile, France) and eluted in 60 µl. According to the ResistanceFinder Instructions for use, 10 µl of the extract was used as input. The pre-amplification step was performed on a SimpliAmp thermal cycler (Thermo Fisher Scientific, Waltham, USA) and the 2SMART step on LightCycler 480 II (Roche, Basel, Switzerland). Of each collected urine sample, 1 mL was stored at − 70 degrees Celsius and transported to Pathofinder to perform the ResistanceFinder 2SMART multiplex PCR.

### Data analysis

The results from the Rapid ESBL NP^®^ test and the ResistanceFinder 2SMART multiplex PCR were compared to the gold standard of culture-based AST with possible ESBL confirmation test results. SPSS version 29.0.0.0 was used to calculate the sensitivity, specificity, positive predictive value (PPV) and negative predictive value (NPV) of both RATs, including the 95% confidence intervals (CI). The PPV and NPV were also corrected for the estimated prevalence of ESBL in the Netherlands. The prevalence of ESBL in *E. coli and K. pneumoniae* isolates from urine samples in inpatient departments excluding the intensive care units in the Netherlands in 2024, was 6.9% and 12.2% respectively [12]. Using these numbers, and the ratio of *E. coli and K. pneumoniae* in our dataset, we estimated the overall prevalence of ESBL to be 7.9%.

### Ethical statement

Urines were collected as part of routine clinical care. The results of the experimental tests were not reported or used for clinical management. Urine samples of patients who explicitly objected to the use of residual material for research were not used in this study. Therefore, the ethical committees of Rijnstate and Radboudumc waived the need for consent and approved this research. All work was conducted in accordance with the Declaration of Helsinki. Data are available upon request.

## Results

During a five-month period (September 2023 – January 2024) a total of 218 urine samples from 218 patients were retrieved from the medical microbiology laboratories of two Dutch hospitals (Rijnstate and Radboudumc). Of the retrieved samples, 66 had dominant growth of ESBL-producing Enterobacterales, 102 contained third generation cephalosporin susceptible Enterobacterales and 50 did not have clinically significant growth after standard incubation of 18 ± 2 h at 35 °C. These 50 samples either contained no growth or contained urogenital flora without the suspicion of uropathogens. Of the 218 samples tested, 122 (56.0%) came from female patients. The average age of the patients was 64 years (range 18–94). Most culture-positive urine samples (84.5%) contained one species of Enterobacterales, with *E. coli* being most prevalent (55.5%), followed by *K. pneumoniae* (12.4%). Table 1 gives an overview of the patients and urine culture results.

**Table 1:** Patient and urine culture characteristics

Characteristics	Total		CUlture +, ESBL +		CULTURE +, ESBL –		Culture -		
Total, n (%)	218	(100.0)	66	(30.3)	102	(46.8)	50	(22.9)	
Female sex, n (%)	122	(56.0)	41	(33.6)	53	(43.4)	28	(22.9)	
Age, years									
18–65, n (%)	99	(45.4)	33	(33.3)	30	(30.3)	36	(36.4)	
>65, n (%)	119	(54.6)	33	(27.7)	72	(60.5)	14	(11.8)	
Hospital									
Radboudumc, n (%)	66	(30.3)	19	(28.8)	34	(51.5)	13	(19.7)	
Rijnstate, n (%)	152	(69.7)	47	(30.9)	68	(44.7)	37	(24.3)	
Number of species of bacteria present									
0, n (%)	50	(22.9)	0	(0.0)	0	(0.0)	50	(100.0)	
1, n (%)	142	(65.1)	51	(35.9)	91	(64.1)	0	(0.0)	
2, n (%)	26	(11.9)	15	(57.7)	11	(42.3)	0	(0.0)	
Species of bacteria									
*Escherichia coli*, n (%)	121	(55.5)	49	(40.5)	72	(59.5)	0	(0.0)	
*Klebsiella pneumoniae*, n (%)	27	(12.4)	13	(48.1)	14	(51.9)	0	(0.0)	
*Proteus mirabilis*, n (%)	9	(4.1)	1	(11.1)	8	(88.9)	0	(0.0)	
*Enterobacter cloacae complex*, n (%)	8	(3.7)	3	(37.5)	5	(62.5)	0	(0.0)	
*Klebsiella oxytoca*, n (%)	2	(0.9)	0	(0.0)	2	(100.0)	0	(0.0)	
*Citrobacter freundii complex*, n (%)	1	(0.5)	0	(0.0)	1	(100.0)	0	(0.0)	
*Citrobacter koseri complex*, n (%)	2	(0.9)	0	(0.0)	2	(100.0)	0	(0.0)	
*Raoultella ornithinolytica*, n (%)	1	(0.5)	1	(100.0)	0	(0.0)	0	(0.0)	

### Rapid ESBL NP^®^ test

Sixteen (16/218: 7.3%) of the Rapid ESBL NP^®^ tests were non-interpretable. These were fairly equally distributed across the groups with 3 (4.5%) from samples with ESBL producing bacteria, 9 (8.8%) from culture positive but ESBL negative samples and 4 (8.0%) from culture negative samples and were all associated with heavily cloudy urine samples. Table 2 shows the outcome of the Rapid ESBL NP^®^ test compared to the AST with possible ESBL confirmatory testing. The sensitivity, specificity, PPV and NPV of the Rapid ESBL NP^®^ test can be found in Table 3, along with the 95% CI. The non-interpretable results were excluded from this analysis.

**Table 2:** The outcomes of the Rapid ESBL NP^®^ test compared to the culture-based AST

	Culture-based AST results			
		Culture +, ESBL +	Culture +, ESBL -	Culture -
Rapid ESBL NP^®^ test	ESBL +	61	0	0
	ESBL -	2	93	46
	NI	3	9	4

**Table 3:** Sensitivity, specificity, positive predictive value and negative predictive value of the Rapid ESBL NP^®^ test. 95% confidence intervals (CI) can be found in brackets

	Rapid ESBL NP^®^ test	
Sensitivity [CI], (%)	96.8	[89.0–99.6]
Specificity [CI], (%)	100	[97.4–100]
PPV [CI], (%)	100	[94.1–100]
NPV [CI], (%)	98.6	[94.7–99.6]
cPPV [CI], (%)	100	[94.1–100]
cNPV [CI], (%)	99.7	[99.0–99.9]

cPPV: corrected PPV. cNPV: corrected NPV. Corrected for an ESBL prevalence of 7.9% in the Dutch population

### ResistanceFinder 2SMART multiplex PCR

The ResistanceFinder 2SMART multiplex PCR showed a variety of resistance genes in our samples (Table 4). No BEL ESBL genes were found. Almost all ESBL positive samples contained CTX-M genes. Some samples contained other genes and combinations thereof, such as TEM, SHV, VEB and PER. TEM and SHV were often found in culture positive samples without ESBL activity. Less than half of the samples with Enterobacterales susceptible for third generation cephalosporins had no resistance genes. If resistance genes were found in culture negative samples, they were most often TEM or SHV (11/50: 22.0%). No resistance genes were found in 66.0% of negative cultures. Table 5 shows how often certain ESBL genes were found in urine samples with specific micro-organisms

**Table 4:** Genes detected by the ResistanceFinder in our samples

Resistance genes detected	Culture +, ESBL + (*n* = 66)		Culture +, ESBL – (*n* = 102)		Culture – (*n* = 50)	
No resistance genes (%)	0	(0.0)	40	(39.2)	33	(66.0)
ESBL genes						
CTX-M (%)	34	(51.5)	4	(3.9)	0	(0.0)
TEM (%)	2	(3.0)	20	(19.6)	7	(14.0)
SHV (%)	0	(0.0)	19	(18.6)	3	(6.0)
PER (%)	0	(0.0)	1	(1.0)	2	(4.0)
VEB (%)	1	(1.5)	0	(0.0)	0	(0.0)
CTX-M, TEM (%)	12	(18.2)	2	(2.0)	0	(0.0)
CTX-M, SHV (%)	8	(12.1)	1	(1.0)	0	(0.0)
TEM, SHV (%)	1	(1.5)	3	(2.9)	4	(8.0)
TEM, PER (%)	0	(0.0)	1	(1.0)	0	(0.0)
CTX-M; SHV; TEM (%)	8	(12.2)	2	(2.0)	0	(0.0)

**Table 5:** ESBL genes found per micro-organism

	*E. coli (n= 121)*		*K.pneumoniae (n= 27)*		Other (*n* = 23)		Culture negative (*n* = 50)	
CTX-M (%)	53	(43.9)	13	(48.1)	2	(8.7)	0	(0.0)
SHV (%)	15	(12.4)	26	(96.2)	1	(4.3)	7	(14.0)
TEM (%)	38	(31.4)	9	(33.3)	2	(8.7)	11	(22.0)
PER (%)	2	(1.7)	0	(0.0)	0	(0.0)	2	(4.0)
VEB (%)	0	(0.0)	0	(0.0)	1	(4.3)	0	(0.0)

The diagnostic performance of the ResistanceFinder 2SMART multiplex PCR differs depending on which target genes are considered ESBL positive. Therefore, we used the following combinations: all ESBL genes; CTX-M, PER and VEB; CTX-M and VEB; and only CTX-M. The calculated diagnostic characteristics using these various combinations of ESBL genes can be found in Table 6.

**Table 6:** Sensitivity, specificity, positive predictive value and negative predictive value for various combinations of (possible) ESBL genes found by the ResistanceFinder 2SMART multiplex PCR compared to the culture-based AST. 95% confidence intervals (CI) can be found in brackets. The combination of (possible) ESBL genes with the highest diagnostic performance is highlighted in bold

Genes ESBL positive	Result ResistanceFinder	Results AST		Sensitivity [CI], %	Specificity [CI], %	PPV [CI], %	NPV [CI], %	cPPV [CI], %	cNPV [CI], %
		ESBL + (*n* = 66)	ESBL – (*n* = 152)						
CTX-M, TEM, SHV, PER, VEB	ESBL +	66	69	100 [94.6–100]	54.6 [46.3–62.7]	48.9 [44.6–53.2]	100 [61.7–74.5]	15.9 [13.7–18.4]	100 [95.7–100]
	ESBL -	0	83						
CTX-M, PER, VEB	ESBL +	63	13	95.5 [87.3–99.1]	91.5 [85.8–95.4]	82.9 [74.2–89.1]	97.9 [93.9–99.3]	48.9 [36.2–61.8]	99.6 [98.7–99.9]
	ESBL -	3	139						
CTX-M, VEB	**ESBL +**	**63**	**9**	**95.5** **[87.3–99.1]**	**94.1** **[89.1–97.3]**	**87.5** **[78.8–93.0]**	**98.0** **[94.0–99.3]**	**58.0** **[42.3–72.3]**	**99.6** **[98.8–99.9]**
	**ESBL -**	**3**	**143**						
CTX-M	ESBL +	62	9	93.9 [85.2–98.3]	94.1 [89.1–97.3]	87.3 [78.5–92.9]	97.3 [93.3–98.9]	57.6 [41.9–72.0]	99.5 [98.6–99.8]
	ESBL -	4	143						

cPPV: corrected PPV. cNPV: corrected NPV. Corrected for an ESBL prevalence of 7.9% in the Dutch population

## Discussion

RATs hold promise to rationalise the use of broad-spectrum antibiotics like carbapenems. In this study, we retrospectively looked at urine samples of patients presenting at two Dutch hospitals. This study aimed to compare the diagnostic characteristics of the phenotypic Rapid ESBL NP^®^ test and a prototype molecular ResistanceFinder 2SMART multiplex PCR to the currently used culture-based AST with possible phenotypical confirmation of ESBL activity. Although our current culture-based AST methods may not be the most sensitive for detecting ESBL in low concentrations, we specifically chose to use the urine culture results of our laboratories to determine whether the RATs could reach the same conclusions, since these culture results are the current standard practice to base the antibiotic treatment of patients suspected of having a UTI on. More sensitive methods for the detection of ESBL in low concentrations, such as sequencing, are not part of routine practice. Detection of lower concentrations of ESBL enzymes or genes may also lead to an increase in the use of carbapenem in situations where this ESBL presence or carriage is not associated with the most prevalent bacterium and therefore may not be the main cause of the UTI. To validate how well these RATs detect lower concentrations of ESBL-producing bacteria, a reference other than standard urine culture should be used. For the Rapid ESBL NP^®^ test, we found a sensitivity and specificity of 96.8% and 100%, respectively, when excluding the 16 non-interpretable results. These results align well with the sensitivity and specificity reported by the manufacturer (93.9% and 98.5%, respectively), even though we used urine directly instead of bacterial isolates [6]. Another study used the similar Rapid ESBL NDP test using urine samples directly (*n* = 450, of which 51 ESBL positive) and found a sensitivity and specificity of 94.1% and 99.8% respectively, when non-interpretable results (1.3%) were considered negative [10]. A recent study used the Rapid ESBL NP^®^ test directly on 274 urine samples, of which 67 contained ESBL-producing bacteria [7]. Here, a sensitivity 86.6% (95% CI: 76.0–93.7) and a sensitivity of 100% (95% CI: 98.2–100), respectively, was found. Studies using the Rapid ESBL NP^®^ test on bacterial isolates report sensitivities between 76% and 100%, and specificities between 81% and 100% [13–20]. Our study aligns with the upper end of these intervals.

Although the Rapid ESBL NP^®^ test was devised for testing bacterial isolates, this study shows a similar performance when used directly on urine. Using urine pellet results in turn-around times of approximately one hour, which is substantially shorter than the 48–72 h the urine culture and subsequent ESBL confirmatory testing take. Also, the Rapid EBSL NP^®^test is relatively simple to implement in clinical practice. A limitation of the test is the number of non-interpretable results. Sixteen (7.3%) of all Rapid ESBL NP^®^ tests were non-interpretable, which is more than previously reported (1.3% [10]). Non-interpretability of the tests was not related to specific outcomes. However, all urine samples which gave non-interpretable tests were notably very cloudy and seemed to have difficulties mixing properly with the red phenol solutions in the test. We conducted additional tests to determine the effect of filtering on the interpretability of cloudy urine samples. The methods and results of this additional experiment can be found in Appendix 1. In conclusion, filtering cloudy urine samples prior to performing the Rapid ESBL NP^®^ test ensures interpretable results. However, we needed 6mL of urine to obtain the 4.5mL of filtered urine necessary to perform the test. In both hospitals in this study, a maximum of 6mL of urine is stored after performing routine diagnostic testing. This is insufficient to re-perform the Rapid ESBL NP^®^ test if the original test is non-interpretable. Moreover, filtering using filter paper adds approximately 20 min to the time it takes to perform the Rapid ESBL NP^®^ test, making the total time of the test approximately 1.5 h. The amount of urine needed for filtering prior to the Rapid ESBL NP^®^ test is an issue easily solved when implementing the test into clinical practice by sending another tube of urine to the lab when a physician orders the test. The additional time filtering takes is unfortunate, but we believe that an extra 20 min is acceptable to prevent non-interpretable results and therefore preserve the opportunity to switch to more appropriate antibiotics earlier.

The prototype version of the ResistanceFinder 2SMART multiplex PCR used in this study detects ESBL genes irrespective of their expression level and can even detect ESBL genes at low bacterial loads. This could explain the lower corrected positive predictive value relative to culture as gold standard. In addition, this prototype was designed to tolerate certain single-nucleotide polymorphisms, enabling detection of both ESBL-encoding and closely related non-ESBL TEM and SHV genes. As expected, since the PCR cannot differentiate between narrow-spectrum and ESBL TEM and SHV variants, these genes were often found in culture positive samples without ESBL activity. In our dataset, CTX-M was the most prevalent type of ESBL, which is in line with other literature, both Dutch [4] and international [21]. The presence of CTX-M was also often associated with a positive ESBL confirmation test (Table 6). Diagnostic performance of the ResistanceFinder varied depending on which gene families were considered ESBL positive, with the best performance observed when only CTX-M and VEB genes were considered ESBL positive. As the ResistanceFinder currently cannot distinguish between narrow-spectrum and ESBL TEM and SHV variants, it would be best to omit these targets if the ResistanceFinder were to be implemented into our clinical practice. The high negative predictive value makes the test a suitable ‘rule-out’ test, where physicians can safely de-escalate when the ResistanceFinder does not find any ESBL genes. The incidence of VEB and PER genes in our dataset was too low to clearly conclude whether or not they should be included as targets. An improved version of ResistanceFinder, with refined gene target interpretation and reporting logic, is currently in development.

A limitation of our methodology is that we retrospectively selected urine samples in this study using the results of the culture-based AST. In clinical practice, culture and AST results will not be known when deciding to perform a rapid test for the detection of antibiotic resistance. Therefore, the diagnostic characteristics found in this study may not be representative of the ‘real-life’ clinical situation, especially as studies using urine samples directly have all used a minimum of 10^5^CFU/mL [7, 10]. A prospective clinical validation study using inclusion criteria that a clinician can use in practice would be the next step towards the clinical validation of these rapid tests, as such a study would most likely also include urine samples with a lower CFU/mL.

To conclude, both the Rapid ESBL NP^®^ test and the ResistanceFinder 2SMART multiplex PCR show a high sensitivity and specificity for the detection of ESBL-producing bacteria in urine samples. The high corrected negative predictive value of both tests ensures suitability to rule out the presence of ESBL. The low corrected positive predictive value of the ResistanceFinder 2SMART multiplex PCR could lead to an increased overuse of carbapenem, if implemented into clinical practice. The high corrected positive predictive value of the Rapid ESBL NP^®^ test also makes this a useful tool to rule in the presence of ESBL. Using Rapid ESBL NP^®^ test could lead to earlier appropriate antibiotic treatment within just over an hour, though a prospective validation study and clinical trial are necessary prior to clinical implementation.

## Figures and Tables

**Fig. 1:**
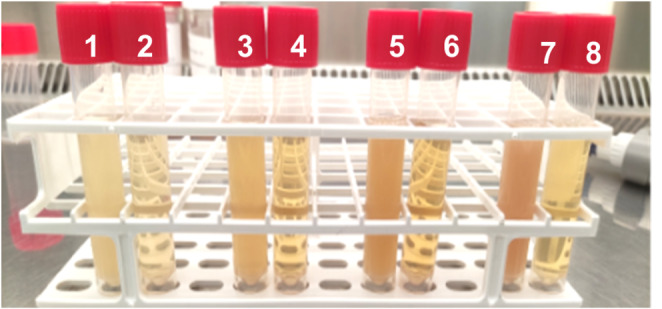
Four sets of urine samples with varying degrees of cloudiness. The left of each set is unfiltered, and the right of each set is the filtered version of the same sample

**Fig. 2:**
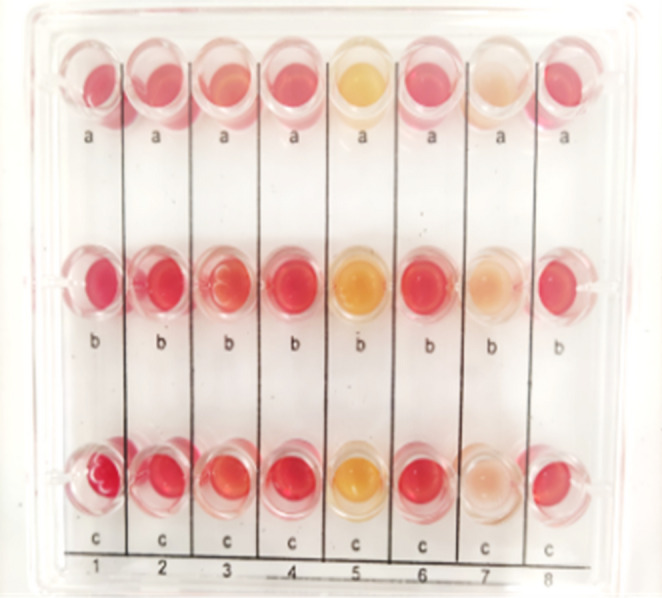
The results of the Rapid ESBL NP^®^ test using the samples from Fig. 1, in the same order. Samples 5 and 7 were cloudy enough to produce a non-interpretable result. Filtering these samples resulted in interpretable results (i.e., ESBL negative in columns 6 and 8), which were in agreement with the outcomes of the AST. Conditions are: top row (**a**) is control well without antibiotic; middle row (**b**) is well with cefotaxime; bottom row (**c**) is well with cefotaxime+tazobactam

## Data Availability

The datasets generated during the current study are not publicly available due to privacy reasons, but are available from the corresponding author upon request.

## Appendix

**Filtration of heavily cloudy urine samples**

All urine samples with non-interpretable test results were notably cloudy. We hypothesized that filtration may ensure interpretable results. Therefore, we filtered additional cloudy urine samples using filter paper with a pore size of 8 μm prior to performing the Rapid ESBL NP^®^ test. We did not filter the original cloudy urines in this dataset due to a lack of material. In seven urine samples, we performed the Rapid ESBL NP^®^ test with and without the filtering step. Of these urine samples, three were sufficiently cloudy to produce non-interpretable results (for example, Figs. 1 and 2, samples 5 and 7). All tests were interpretable after filtering (Figs. 1 and 2, samples 6 and 8). The results were in agreement with the results of the urine cultures. To determine whether bacteria were able to pass through the filter, we performed the Rapid ESBL NP^®^ test on four filtered ESBL positive urine samples with growth of at least 10^5^ CFU/mL. All these four tests gave a positive result after filtering. No cloudy ESBL positive samples were found with a sufficient volume to perform the test on an unfiltered and filtered version of the same sample.
